# Supplementary material for: Creating functional groups of marine fish from categorical traits
Source: PeerJ. 2018 Oct 23;6:e5795. doi: 10.7717/peerj.5795 (PMC6202955; doi:10.7717/peerj.5795)
Supplement: Table S1 [file peerj-06-5795-s001.pdf]

TABLE S1 LIST OF THE POSSIBLE TRAITS TO INCLUDE

Table 1. List of diet and life history traits to include in clustering

Trait	References	Measurement type
Diet		
Primary diet category	Defined by authors	Categorical - Nominal
Food type/ properties	Sibbing & Nagelkerke 2000, Reecht et al., 2013	Categorical - Nominal
Life History		
Number of eggs	Bremner et al. 2006a, Clavel et al. 2013, Gravel et al. 2016	Numerical - Integer
Number of spawns per year	Clavel et al. 2013	Numerical - Integer
Reproductive season	NEW	Categorical - Nominal
Parental care	Franco et al. 2008, Gravel et al. 2016	Categorical - Nominal
Reproductive mode	Bremner et al. 2006a, Elliott et al. 2007, Franco et al. 2008	Categorical - Nominal
Sexual differentiation	Bremner et al. 2006a	Categorical - Nominal
Phylogenetic diversity	Froese & Pauly 2017	Numerical - Integer
Population doubling	Bremner et al. 2006a, Froese & Pauly 2017	Categorical - Nominal
Vulnerability	Froese & Pauly 2017	Numerical
Gregarious/ Schooling type	Bremner et al. 2006a, Stuart-Smith et al. 2013, Spitz et al. 2014	Categorical - Nominal
Seasonal migration	Bremner et al. 2006a, Spitz et al. 2014	Categorical - Nominal
Diel migration	Bremner et al. 2006a, Stuart-Smith et al. 2013, Spitz et al. 2014	Categorical - Nominal
Ontogenetic migration	Reum & Essington 2008	Categorical - Nominal
Maximum age	Bremner et al. 2006a, Clavel et al. 2013, Gravel et al. 2016	Numerical - Integer
Mortality	Froese & Pauly 2017	Numerical - Continuous
Age at maturity	Bremner et al. 2006a, Clavel et al. 2013	Numerical - Continuous
Length at maturity	Bremner et al. 2006a, Clavel et al. 2013	Numerical - Continuous

Table 2. List of morphological and habitat traits to include in clustering

Trait	References	Measurement type
Morphological traits		
Body length (max)	Winemiller & Rose 1992, Froese & Binohlan 2000, Sibbing & Nagelkerke 2000, Boyle & Horn 2006, Bremner et al. 2006b, Clavel et al. 2013, Spitz et al. 2014, Gravel et al. 2016, Mindel et al. 2016	Numerical - Continuous
Body mass (max)	Palomares & Pauly 1989, Dumay et al. 2004, Reecht et al. 2013, Spitz et al. 2014, Gravel et al. 2016	Numerical - Continuous
Growth rate	Bremner et al. 2006b	Numerical - Continuous
Swimming mode	Sfakiotakis et al. 1999	Categorical - Nominal
Dorsal fin shape	Standen & Lauder 2005	Categorical - Nominal
Caudal fin shape	Roberts 1987, Bridge et al. 2016, Gravel et al. 2016	Categorical - Nominal
Pelvic fin position	Lauder & Drucker 2004	Categorical - Nominal
Body shape/form	Webb 1984, Roberts 1987, Spitz et al. 2014, Gravel et al. 2016	Categorical - Nominal
Eye position	Clavel et al. 2013, Mindel et al. 2016	Categorical - Nominal
Barbel presence	Piet 1998, Reecht et al. 2013, Gravel et al. 2016	Categorical - Nominal
Oral gape position	Roberts 1987, Sibbing & Nagelkerke 2000, Ward-Campbell et al. 2005, Boyle & Horn 2006, Reecht et al. 2013, Gravel et al. 2016	Categorical - Nominal
Shape of teeth	Roberts 1987, Gravel et al. 2016	Categorical - Nominal
Number of gill rakers	Sibbing & Nagelkerke 2000, Boyle & Horn 2006	Numerical - Integer
Habitat		
Depth found	Spitz et al. 2014, Froese & Pauly 2017	Numerical - Continuous
Preferred temperature	Froese & Pauly 2017	Numerical - Integer
Temperature type	Froese & Pauly 2017	Categorical - Nominal
Horizontal habitat	Spitz et al. 2014	Categorical - Nominal
Vertical habitat	Sibbing & Nagelkerke 2000, Bremner et al. 2006a, Spitz et al. 2014	Categorical - Nominal
Estuarine use	Elliott et al. 2007, Franco et al. 2008	Categorical - Nominal

References

Boyle KS, Horn MH. 2006. Comparison of feeding guild structure and ecomorphology of intertidal fish assemblages from central California and central Chile. Marine Ecology Progress Series 319:65-84

Bremner J, Paramor OAL, Frid CLJ. 2006a. Developing a methodology for incorporating ecological structure and functioning into designation of Special Areas of Conservation (SAC) in the 0-12 nautical mile zone.

Bremner J, Rogers SI, Frid CLJ. 2006b. Matching biological traits to environmental conditions in marine benthic ecosystems. Journal of Marine Systems 60:302-316

Bridge TCL, Luiz OJ, Coleman RR, Kane CN, Kosaki RK. 2016. Ecological and morphological traits predict depth-generalist fishes on coral reefs. Proceedings of the Royal Society B 283:1823

Clavel J, Poulet N, Porcher E, Blanchet S, Grenouillet G, Pavoine S, Biton A, Seon-Massin N, Argillier C, Daufresne M, Teillac-Deschamps P, Julliard R. 2013. A new freshwater biodiversity indicator based on fish community assemblages. PLoS One 8:e80968

Dumay O, Tari PS, Tomasini JA, Mouillot D. 2004. Functional groups of lagoon fish species in Languedoc Roussillon, southern France. Journal of Fish Biology 64:970-983

Elliott M, Whitfield AK, Potter IC, Blaber SJM, Cyrus DP, Nordlie FG, Harrison TD. 2007. The guild approach to categorizing estuarine fish assemblages: a global review. Fish and Fisheries 8:241-268

Franco A, Elliott M, Franzoi P, Torricelli P. 2008. Life strategies of fishes in European estuaries: the functional guild approach. Marine Ecology Progress Series 354:219-228

Froese R, Binohlan C. 2000. Empirical relationships to estimate asymptotic length, length at first maturity and length at maximum yield per recruit in fishes, with a simple method to evaluate length frequency data. Journal of Fish Biology 56:758-773

Froese R, Pauly D. 2017. FishBase. Accessed 02-02-2017.

Gravel D, Albouy C, Thuiller W. 2016. The meaning of functional trait composition of food webs for ecosystem functioning. Philosophical Transactions of the Royal Society B 371:1694

Lauder GV, Drucker EG. 2004. Morphology and experimental hydrodynamics of fish fin control surfaces. IEEE Journal Ocean Engineering 29:556-571

Mindel BL, Webb TJ, Neat FC, Blanchard JLZ. 2016. A trait-based metric sheds new light on the nature of the body size–depth relationship in the deep sea. Journal of Animal Ecology 85:427-436

Palomares ML, Pauly D. 1989. A multiple regression model for prediction the food consumption of Marine Fish populations. Marine Freshwater Research 40:259-273

Piet GJ. 1998 Ecomorphology of a size-structured tropical freshwater fish community. Environmental Biology of Fishes 51:67-86

Reecht Y, Rochet MJ, Trenkel VM, Jennings S, Pinnegar JK. 2013. Use of morphological characteristics to define functional groups of predatory fishes in the Celtic Sea. Journal of Fish Biology 83:355-377

Reum JCP, Essington TE. 2008. Seasonal variation in guild structure of the Puget-Sound demersal fish community. Estuaries and Coasts 31:790-801

Roberts CD. 1987. First records of demersal fishes from the North Cape three kings area of New Zealand, with a record of a prespawning aggregation of ling, Genypterus blacodes (note). New Zealand Journal of Marine Freshwater Research 21:157-161

Sfakiotakis M, Lane DM, Davies JBC. 1999. Review of fish swimming modes for aquatic locomotion. IEEE Journal of Ocean Engineering 24:237-252

Sibbing FA, Nagelkerke LAJ. 2000 Resource partitioning by Lake Tana barbs predicted from fish morphometrics and prey characteristics. Reviews in Fish Biology and Fisheries 10:393-437

Spitz J, Ridoux V, Brind'Amour A. 2014. Let's go beyond taxonomy in diet description: testing a trait-based approach to prey–predator relationships. Journal of Animal Ecology 83:1137-1148

Standen EM, Lauder GV. 2005. Dorsal and anal fin function in bluegill sunfish Lepomis macrochirus three-dimensional kinematics during propulsion and manoeuvring. Journal of Experimental Biology 208:2753-2753

Stuart-Smith RD, Bates AE, Lefcheck JS, Duffy JE, Baker SC, Thomson RJ, Stuart-Smith JF, Hill NA, Kininmonth SJ, Airoldi L, Becerro MA, Campbell SJ, Dawson TP, Navarrete SA, Soler GA, Strain EMA, Willis TJ, Edgar GJ. 2013. Integrating abundance and functional traits reveals new global hotspots of fish diversity. Nature 501:539-542

Ward-Campbell BMS, Beamish FWH, Kongchaiya C. 2005. Morphological characteristics in relation to diet in five coexisting Thai fish species. Journal of Fish Biology 67:1266-1279

Webb PW. 1984. Form and function in fish swimming. Scientific American 251:72-82

Winemiller KO, Rose KA. 1992. Patterns of Life-history diversification in North American fishes: implications for population regulation. Canadian Journal of Fisheries Aquatic Science 49:2196-2218
