# Supplementary material for: Creating functional groups of marine fish from categorical traits
Source: PeerJ. 2018 Oct 23;6:e5795. doi: 10.7717/peerj.5795 (PMC6202955; doi:10.7717/peerj.5795)
Supplement: Data S1 [file peerj-06-5795-s002.pdf]

DATA S1 MISSING DATA IMPUTATION METHODS

Imputation using random forests

The first approach uses random forests to impute missing values and was implemented in the R package `missForest` which can impute continuous, categorical and mixed data. Random forests do not have any assumptions of underlying distributions, can accommodate high dimension data and can handle complex interactions and non-linear relationships among variables (Stekhoven and Bühlmann, 2012). This package was chosen as it does not have any assumptions of the underlying distributions of the traits, can deal with mixed data and does not require tuning of the parameters (Stekhoven and Bühlmann, 2012). To estimate the unknown variables, `missForest` fits a random forest to the observed part of each variable with missing data and uses the trained model to predict the missing data. To create a random forest, many decision trees are trained with each tree only seeing a random subset of the data. Decision trees classify the data one variable at a time, where the variable becomes the node and the data separated into groups based on the nodes criteria. To create the final forest, all of the trees are averaged to generate output probabilities. The random forest was implemented using the R package `randomForest` (Liaw and Wiener, 2002).

Imputation using Multiple Correspondence Analysis (MCA)

`missMDA` (Josse and Husson, 2016) uses iterative MCA algorithms, which consists of an initialization step in which missing values in the indicator matrix are imputed by initial values, such as the proportion of the category. This initialization for categorical variables is equivalent to mean imputation for continuous variables. Then, MCA is performed on the imputed indicator matrix to obtain an estimation of parameters, and missing values are imputed using the fitted values.

Imputation using Multivariate Imputation by Chained Equations (MICE)

`MICE` is a popular R data imputation package that offers a range of methods to impute data (van Buuren and Groothuis-Oudshoorn, 2011). For our purposes we used the option from `MICE` that handles factor variables with more than 2 categories. `MICE` imputes data in three steps. First, plausible values are drawn from a distribution that is specifically modelled for each missing value. This is important as in other imputation packages and methods it is often assumed that all of the missing values come from the same distribution. Second, m imputed datasets are created based on the missing value distributions. In each of these datasets, the non-missing data remain identical, while imputed values may differ. A value of Q is determined for each dataset which allows the uncertainty in each estimation to be calculated. Finally, estimates of missing data are pooled, and since Q is approximately normally distributed, the mean is imputed as the missing value. Since the estimate comes from a normal distribution we are also able to estimate its variance. For our case, we only have nominal variables, therefore imputing missing data using a normal distribution is inappropriate. Instead a polytomous regression is used (Brand, 1999). This allows the underlying relationships between variables to vary non-linearly. This type of regression can also handle nominal variables with different category levels.

REFERENCES

Brand, J. (1999). *Development, implementation and evaluation of multiple imputation strategies for the statistical analysis of incomplete data sets*. PhD thesis, University of Rotterdam.

Josse, J. and Husson, F. (2016). missmda: a package for handling missing values in multivariate data analysis. *Journal of Statistical Software*, 70(1):1–31.

Liaw, A. and Wiener, M. (2002). Classification and regression by randomforest. *R news*, 2(3):18–22.

Stekhoven, D. J. and Bühlmann, P. (2012). Missforest: non-parametric missing value imputation for mixed-type data. *Bioinformatics*, 28(1):112–118.

van Buuren, S. and Groothuis-Oudshoorn, K. (2011). mice: Multivariate imputation by chained equations in r. *Journal of statistical software*, 45(3):1–68.
