# Supplementary material for: Creating functional groups of marine fish from categorical traits
Source: PeerJ. 2018 Oct 23;6:e5795. doi: 10.7717/peerj.5795 (PMC6202955; doi:10.7717/peerj.5795)
Supplement: Data S2 [file peerj-06-5795-s003.pdf]

DATA S2 DISTANCE MATRIX CALCULATIONS

This section outlines the theory behind and the formulae for each of the distance matrices used. Distance matrices are applied to the matrix $X = [x_{ic}]$ where $i = 1,2,\ldots,n$ (n is the total number of objects); $k = 1,2,\ldots,m$ (m is the total number of variables). The number of categories of the cth variable is denoted as C_c , absolute frequency as f, and relative frequency as p.

- Simple Matching is the simplest approach to creating a distance matrix, awarding 1 to observations that are the same and 0 if not (Eq. 1 Gower, 1967). This is the approach used for Gower's similarity measure of nominal data:

$$S_{SM}(x_{ic},x_{jc}) = \begin{cases} 1 & \text{if} x_{ic} = x_{jc} \\ 0 & \text{otherwise} \end{cases} \tag{1}$$

- Eskin et al. (2002) uses a simple matching criteria where a value of 1 is assigned if the values match and gives more weight to mismatches on variables that have more categories (Eq. 2). Mismatches on binary variables are given the lowest similarity values:

$$S_{ES}(x_{ic},x_{jc}) = \begin{cases} 1 & \text{if } i \text{ and } j \text{ agree on } c \\ \dfrac{n_c^2}{n_c^2+2} & \text{otherwise} \end{cases} \tag{2}$$

where n_c is the number of categories in the $c^t h$ variable.

- Inverse occurrence frequency has the same approach as Eskin but give less weight to mismatches on variables that have more categories. This uses the absolute frequencies of observed categories:

$$S_{IOF}(x_{ic},x_{jc}) = \begin{cases} 1 & \text{if} x_{ic} = x_{jc} \\ \dfrac{1}{1+\log f(x_{ic}) \times f(x_{jc})} & \text{otherwise} \end{cases} \tag{3}$$

- When comparing two observations of a given variable Goodall's measure takes into account relative frequencies of categories (Eq. 4; Goodall, 1966). A similarity value is assigned based on the normalised similarity between the two observations, where the similarity value is higher if it occurs infrequently. This method takes into account that individuals attributes occur stochastically and independently in a population:

$$S_{GD}(x_{ic},x_{jc}) = \begin{cases} 1-\sum_{q\in Q} p^2(q) & \text{if} x_{ic} = x_{jc} \\ 0 & \text{otherwise} \end{cases} , Q \subseteq X_i : \forall q, p(q) \geq (x_{ic}) \tag{4}$$

- Lin is an information theoretic definition of similarity based on relative frequencies (Lin, 1998). Weights are given to both matches and mismatches where more frequent matches get higher weights, and lower weights are given to infrequent categories that mismatch:

$$S_{LN}(x_{ic},x_{jc}) = \begin{cases} 2\ln p(x_{ic}) & \text{if} x_{ic} = x_{jc} \\ 2\ln(p(x_{ic}) + p(x_{jc})) & \text{otherwise} \end{cases} \tag{5}$$

REFERENCES

Eskin, E., Arnold, A., Prerau, M., Portnoy, L., and Stolfo, S. (2002). A geometric framework for unsupervised anomaly detection. In Barbará, D. and Jajodia, S., editors, *Applications of Data Mining in Computer Security*, pages 77–101. Springer US, Boston, MA.

Goodall, D. W. (1966). A new similarity index based on probability. *Biometrics*, 22(4):882–907.

Gower, J. C. (1967). A comparison of some methods of cluster analysis. *Biometrics*, 23(4):623–637.

Lin, D. (1998). An information-theoretic definition of similarity. In *Proceedings of the 15th International Conference on Machine Learning*, volume 98, pages 296–304. Morgan Kaufmann Publishers Inc., Morgan Kaufmann Publishers Inc.
