# Supplementary material for: Creating functional groups of marine fish from categorical traits
Source: PeerJ. 2018 Oct 23;6:e5795. doi: 10.7717/peerj.5795 (PMC6202955; doi:10.7717/peerj.5795)
Supplement: Figure S1 [file peerj-06-5795-s004.pdf]

FIGURE S1 DISCRETIZATION

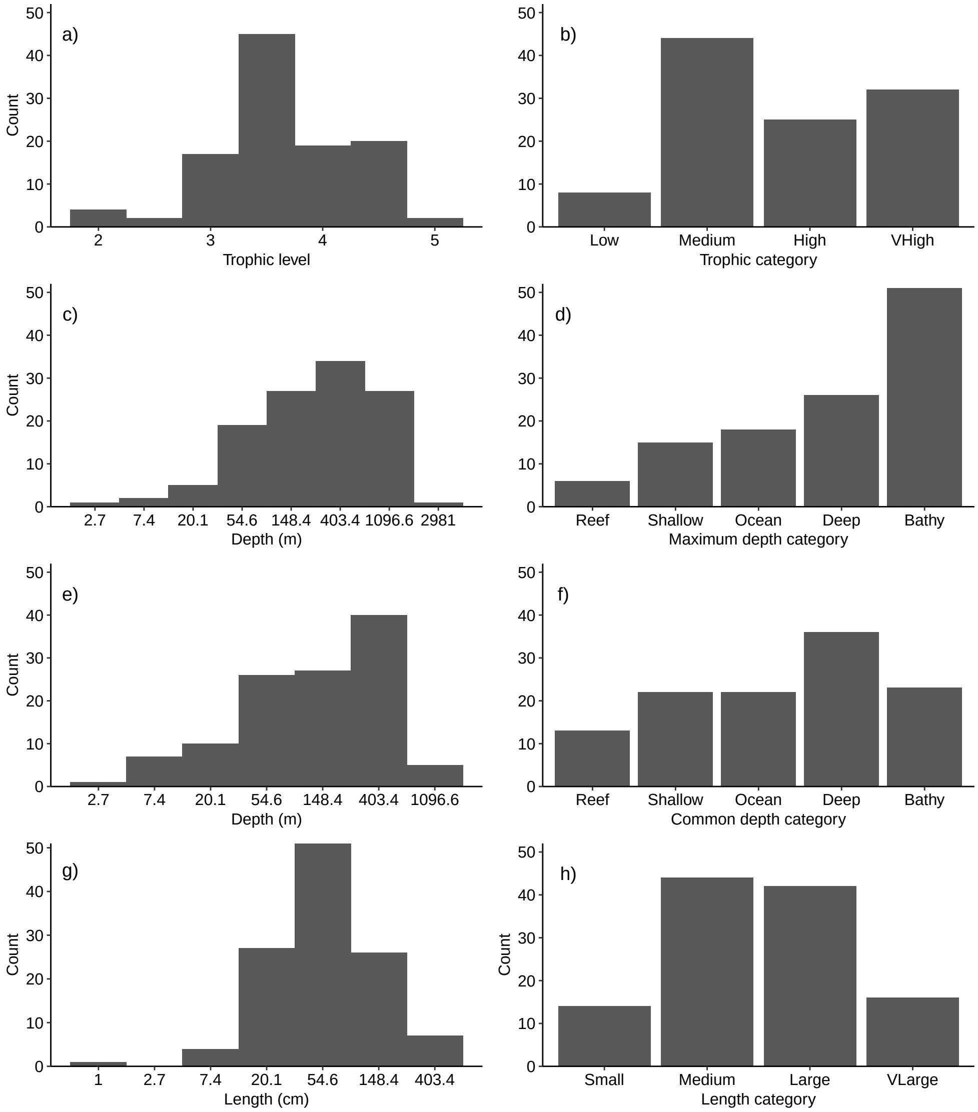

Figure 1. Histograms of the raw data with the corresponding bar plot of the discretized variable for trophic level (a,b), maximum depth (m) (c,d), common maximum depth (m) (e,f) and length (cm) (g,h).
