# Supplementary material for: Creating functional groups of marine fish from categorical traits
Source: PeerJ. 2018 Oct 23;6:e5795. doi: 10.7717/peerj.5795 (PMC6202955; doi:10.7717/peerj.5795)
Supplement: Figure S2 [file peerj-06-5795-s005.pdf]

FIGURE S2 ALTERNATIVE SOLUTION FOR EVALUATING RELIABILITY USING THE GINI COEFFICIENT

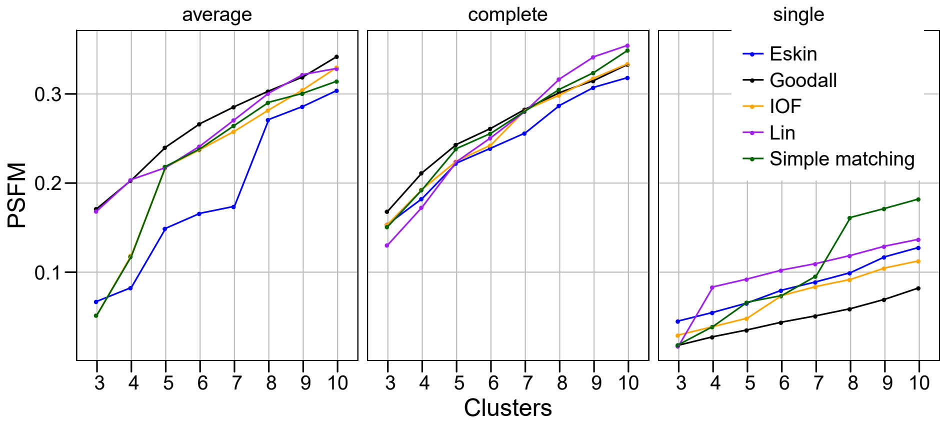

Figure 1. Evaluation of the optimal number of clusters using the pseudo F coefficient based on the mutability (PSFM). All 10 distance matrices (coloured lines) from the *nomclust* package are displayed tested across three clustering algorithms (facets).

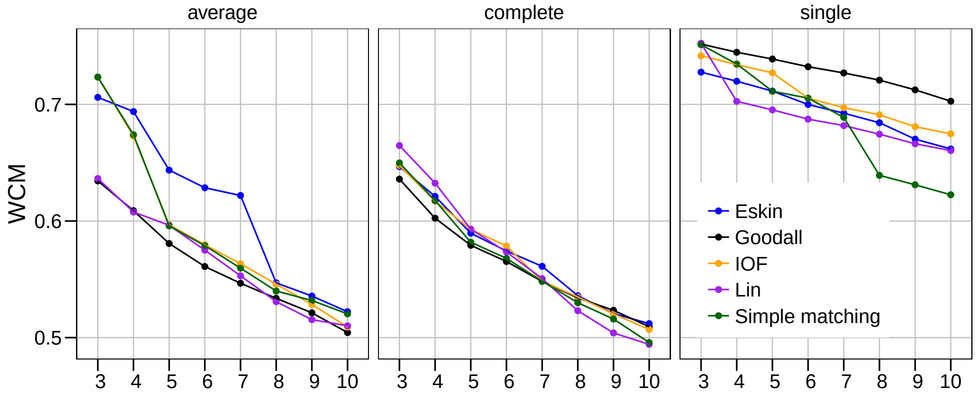

Figure 2. Evaluation of the optimal number of clusters using the within-cluster mutability coefficient (WCE). All 10 distance matrices (coloured lines) from the *nomclust* package are displayed tested across three clustering algorithms (facets).
